# Public’s Health Risk Awareness on Urban Air Pollution in Chinese Megacities: The Cases of Shanghai, Wuhan and Nanchang

**DOI:** 10.3390/ijerph13090845

**Published:** 2016-08-25

**Authors:** Xiaojun Liu, Hui Zhu, Yongxin Hu, Sha Feng, Yuanyuan Chu, Yanyan Wu, Chiyu Wang, Yuxuan Zhang, Zhaokang Yuan, Yuanan Lu

**Affiliations:** 1 School of Public Health, Nanchang University, 461 Bayi Road, Donghu District, Nanchang 330006, China; liuxioajun@email.ncu.edu.cn (X.L.); 416524115699@email.ncu.edu.cn (H.Z.); hyx@ncu.edu.cn (Y.H.); 2 School of Public Health, Fudan University, 138 Yixueyuan Road, Xuhui District, Shanghai 200032, China; 13211020033@fudan.edu.cn; 3 Global Health Institute, Wuhan University, 115 Donghu Road, Donghu District, Wuhan 430071, China; 2014203050033@whu.edu.cn; 4 Department of Public Health Sciences, University of Hawaii at Mānoa, 1960 East-West Road, Honolulu, HI 96822, USA; yywu@hawaii.edu (Y.W.); wcy_james@126.com (C.W.); zhang20050732@126.com (Y.Z.)

**Keywords:** health risk awareness, urban air pollution, public concern, Chinese megacities

## Abstract

This study assessed the public’s health risk awareness of urban air pollution triggered by three megacities in China, and the data are the responses from a sample size of 3868 megacity inhabitants from Shanghai, Nanchang and Wuhan. Descriptive analyses were used to summarize the respondents’ demographics, perceived health risks from air pollution and sources of health-related knowledge on urban air pollution. Chi-square tests were used to examine if participants’ demographics were associated with participant’s general attitudes towards current air quality and the three perceived highest health risks due to urban air pollution. We found low rate of satisfaction of current urban air quality as well as poor knowledge of air pollution related indicator. Participants’ gender, age and travel experience were found to be associated with the satisfaction of current air quality. The knowledge of air pollution related indicator was significantly affected by respondents’ education, monthly income, health status, and sites of study. As many as 46.23% of the participants expressed their feelings of anxiety when exposed to polluted air, especially females, older adults and those with poor health conditions. Most participants believed that coughs/colds, eye problems and skin allergies were the three highest health risks due to urban air pollution based on public education through television/radio, internet and newspaper/magazine. Further public health education is needed to improve public awareness of air pollution and its effects.

## 1. Introduction

Globally air quality has deteriorated seriously in the past few decades with economic development and modern industrialization [1,2,3]. Air pollution and its adverse effects on human health have drawn more global attention since the smog incident in Donora, Pennsylvania in 1948 [4] and the killer London smog event of 1952 [5]. Up to now, air pollution is estimated to cause 3.7 million premature deaths per year worldwide [6]. It has become the leading global risk for public health, and most megacities in developing countries are experiencing the worst air pollution [7]. Urban dwellers in developing countries are at higher risk of cardiovascular diseases, respiratory diseases and other illnesses [8,9,10]. Due to the pressures of social and economic development, many megacities in developing countries are facing more complicated and serious environmental problems and challenges. Air pollution in urban areas is a complex mixture of particles and gas-phase pollutants arising from a myriad of sources. Most megacities in low- and middle-income level countries experience extremely high levels of both visible (particulate matter) and invisible (gases) forms of air pollution [11,12,13], and their dwellers are at the highest risk [14].

There are more than 700 million urban populations in China. The recent migrations and relocation from rural areas to urban have directly resulted in more than 15 megacities with a population of more than 10 million [15]. According to China Environmental Bulletin, only 16 out of the 161 nationally selected cities have met the New Air Quality Standard for Testing launched in 2014 in China, yielding a 9.9% compliance rate [16]. The Organization for Economic Cooperation and Development (OECD) estimated that urban air pollution contributed to about 350,000 to 500,000 deaths per year in China’s major cities [17]. Large scale urbanization in China with unprecedented urban expansion and infrastructure development has led to the release of large amount of harmful pollutants to the atmosphere, seriously threatening people’s health [18,19].

Studies on public environmental health related risk perception originated in developed countries between the 1960s and 1970s, and most recent studies in this area were mainly conducted in Western countries [20,21]. In China, risk management on atmospheric pollution has been one of main important topics for the government [22], as urban air pollution has become a major health hazard to its people. Successful management of environmental risks, especially the urban air pollution risk, can hardly be achieved without the public’s understandings and cooperation [23]. Public perception of air pollution related health risks could also affect their personal environmental habits and behavior, and influence their judgments toward relevant risk management policy in a direct or indirect manner [24,25]. We are interested in evaluating the awareness of urban air pollution-health risk, and this study was designed to identify the main factors associated with the public’s health risk awareness toward urban air pollution, and to explore more effective way for urban air pollution control by bridging the gap between scientific research and public health risk awareness on urban air pollution.

To understand the megacity inhabitants’ health risk awareness of urban air pollution and its potential effect on public health, this study is aimed at assessing the first steps of developing a model of the social dilemma of unsafe air quality in Chinese megacities. In this paper, we evaluated the public’s awareness of health risk of urban air pollution using data from the three megacities along middle and lower reaches of the Yangtze River: Shanghai, Nanchang and Wuhan. The findings will not only have a huge impact on residents’ health-related behavior, but also provide the necessary scientific basis for government in their risk management of urban air pollution and better urban governance in the future [26,27,28].

## 2. Methods

### 2.1. Study Design and Data Collection

The Central Government of China launched the program called *Yangtze River Economic Belt* in 2014, which has accelerated development of middle and lower reaches of the Yangtze River Urban Agglomerations and it is being carried out in full swing. Hence, the three megacities in this area: Shanghai, Nanchang and Wuhan were chosen for this study as the most representative fast growing megacities in China. According to the previous related studies conducted in China [29], this study aimed at investigating the residents’ health risk awareness on urban air pollution in China’s megacities. The target populations were adults aged 18 years and above and have lived in the three megacities for at least one year. The qualified individuals were briefly introduced about this study and asked if they were willing to participate. Utilizing a face-to-face interview style, data was collected in parks, pedestrian streets, and shopping centers where the population density is high in each study site. The study was conducted by trained public health graduates from Fudan University, Wuhan University and Nanchang University during the spring and summer time of 2015. Each survey took five to eight minutes to complete.

### 2.2. Questionnaire

The questionnaire used in this study was designed by public health experts from University of Hawaii at Mānoa [29]. The questionnaire focused on public’s health risk awareness on urban air pollution in Chinese megacities. Respondents were asked to provide their home address at the beginning of the questionnaire in order to avoid repeated participation. Four subject areas of the survey questions were included: (1) social demographic characteristics including participants’ gender, age, educational level, monthly income, self-reported health status and travel experience; (2) attitudes towards current air quality in Chinese megacities, including participants’ satisfaction of current air quality, knowledge of air pollution related indicator (API) and anxious feelings when exposed to contaminated air; (3) participants’ beliefs about the high health risks due to urban air pollution, including coughs/colds, eye problems, difficulty breathing, asthma, etc.; (4) where the respondents get health-related knowledge on urban air pollution, including doctor/physician, friend or family member, television or radio and internet sites, etc.

### 2.3. Quality Control

The questionnaire was designed based on the local circumstances and the specific objectives of this study by public health professors of Fudan University, Wuhan University and Nanchang University. Qualified and trained graduate students from these three universities conducted the survey and collected the data. Pretest surveys among members of the target group were conducted to ensure face validity and comprehension of the survey, and the questionnaires were verified on site for quality control. Completed questionnaires were organized and numbered. Unqualified questionnaires, such as one questionnaire with all identical answers or with extensive missing answers, were excluded from the analysis. EpiData3.1 (Odense, Denmark) was used to set up the database, and logical verification of the questionnaires was done to ensure the accuracy of the data. Data were entered twice separately by two students into two different files and was cross-checked. Discordant information was verified by comparing to the original questionnaire.

### 2.4. Statistical Analysis

Using EpiData3.1, questionnaires were imported into the computer, Excel 2010 (Microsoft Ltd., Washington, DC, USA) was applied to manage data and the Statistical Package for the Social Sciences (SPSS) (SPSS Inc., Chicago, IL, USA) version 18.0 was used for data analysis. Two sided *p*-values of less than 5% were considered to be statistically significant. Descriptive statistics frequencies and proportions were used to summarize the general demographic characteristics, respondents’ perceived health risks of air pollution and sources of health-related knowledge on urban air pollution. Chi-square tests of independence were used to examine if participants’ gender, age, educational level, monthly income, self-reported health status and travel experience were associated with participant’s general attitudes towards current air quality in Chinese megacities and the three perceived highest health risks due to urban air pollution. We also reported the adjusted odds ratios (OR) and 95% confidence interval (CI) obtained from multivariable logistic regression models to identify the main factors that were most predictive of the respondents’ general attitudes towards current air quality. Pearson’s correlation coefficient was adopted to examine the strength and direction of the relationship between respondents’ general satisfaction, anxious feeling and knowledge of API toward current air quality.

### 2.5. Ethical Considerations

The study protocol was reviewed and approved by the Research Ethics Board of University of Hawaii at Mānoa, and local institution protocols followed (CHS#21267). To protect the respondents’ confidentiality, interviews were conducted anonymously with written informed consent. Informed consent information was attached on top of each questionnaire and introduced before the surveys.

## 3. Results

### 3.1. Sample Characteristics

Table 1 summarizes the general characteristics of the participants. A total of 3868 respondents participated in this study, including 1375 respondents from Shanghai, 1207 from Wuhan and 1286 from Nanchang; among which 1997 were male (51.63%) and 1871 female (48.37%). These respondents were classified into four age groups including <25, 25–45, 45–65 and >65, accounting for 23.40%, 33.40%, 29.96% and 13.24% of the total sample respectively. Over 90% of respondents had at least a junior high school education and 42.71% of the participants had monthly incomes ranging from 3000 to 4999 Yuan (RMB). Most of the participants had previous travel experience. Results of Chi-square tests showed that the three study sites varied in education level, monthly income and travel experience.

### 3.2. Respondents’ General Satisfaction of Current Air Quality

The respondents’ general satisfaction of current air quality is summarized in Table 2. More than half of the participants (56.69%) were not satisfied with current air quality. Chi-square test showed that participants’ gender, age and travel experience were associated with general satisfaction of current air quality. Multivariable logistic regression analysis (Table 3) indicated that female, older adults and those who had travel experiences were not satisfied with the current air quality.

### 3.3. Respondents’ Anxious Feelings When Exposed to Contaminated Air

Table 4 reported the relationship between demographic factors and respondents’ anxious feelings when exposed to contaminated air. About 46% of the participants expressed their anxious feelings when exposed to polluted air. Chi-square test illustrated that respondents’ negative feelings when exposed to polluted air is significantly affected by gender (χ^2^ = 5.739, *p* = 0.017), age (χ^2^ = 14.734, *p* = 0.002) and health status (χ^2^ = 61.438, *p* < 0.001). Moreover, Multivariable logistic regression analysis (Table 3) revealed that age 45–65 and above more likely to have anxious feelings compared to age <25 group (age 45–65, OR = 1.48, 95% CI: 1.12–3.25; age >65, OR = 2.09, 95% CI: 2.18–4.22).

### 3.4. Respondents’ Knowledge of Air Pollution Related Indicator (API)

Respondents’ knowledge of the API is summarized in Table 5. The results show that more than half of the respondents (57.50%) didn’t know what the API is. As shown in Table 4, the knowledge of API was significantly affected by respondents’ educational level (χ^2^ = 10.174, *p* = 0.006), monthly income (χ^2^ = 60.627, *p* < 0.001), health status (χ^2^ = 6.060, *p* = 0.014) and different study sites (χ^2^ = 8.610, *p* = 0.013). The results from Multivariable logistic regression analysis (Table 3) indicated that those with higher educational level, higher monthly income and poor self-rated health status population were more likely to pay attention to the API. Moreover, respondents from Shanghai had higher knowledge of API.

The results of Chi-square tests were summarized in Table 2 and Table 3, which identified that the respondents’ general satisfaction of current air quality, anxious feelings when exposed to contaminated air and knowledge of API was significantly related to one another. To better understand how those responses are correlated with one another, Pearson’s correlation coefficient, ϕ was calculated to evaluate the association between respondents’ general satisfaction, anxious feeling toward current air quality and knowledge of API (Table 6). Weak positive relationships were found between respondents’ general satisfaction of current air quality and anxious feelings (ϕ = 0.192, *p* < 0.001), and between respondents’ knowledge of API with general satisfaction of current air quality and anxious feelings (ϕ = 0.091, 0.059 respectively, *p* < 0.01 for both).

### 3.5. Respondents’ Perceived Health Risks from Urban Air Pollution

**Figure 1:**
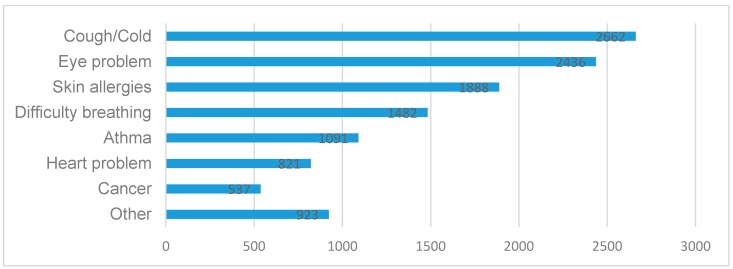
Respondents’ perceived health risks from air pollution: the top three needing more attention.

As shown in Figure 1, most participants responded that the main health risks due to air pollution are cough/cold (2662, 68.82%), eye problem (2436, 62.98%), skin allergies (1888, 48.81%) and difficulty breathing (1482, 38.31%). Table 7 illustrates the connection between respondents’ demographic factors and their beliefs about the three highest health risks due to urban air pollution. Chi-square test confirmed that females are more likely to believe cough/cold (χ^2^ = 5.368, *p* = 0.021), eye problem (χ^2^ = 38.963, *p* < 0.001) and skin allergies (χ^2^ = 8.672, *p* = 0.003) were the most common health risks related to air pollution. Linear trend chi-square test indicated that the older people believed cough/cold (χ^2^ = 44.377, *p* < 0.001) and eye problem (χ^2^ = 6.915, *p* = 0.009) were the most common health risks, while those with higher education level considered skin allergies (χ^2^ = 97.863, *p* < 0.001) was the most common health risk. Chi-square test also revealed that choosing cough/cold (2662, 68.82%), eye problem as the main health risks were significantly associated with respondents’ health status. It is interesting to find that different study sites had different opinions upon whether skin allergies were the main health risks due to air pollution.

### 3.6. Sources of Health-Related Knowledge on Urban Air Pollution for Respondents

**Figure 2:**
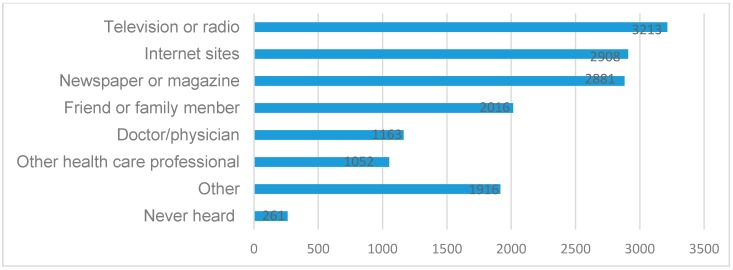
Where the respondents get health-related knowledge on urban air pollution.

Respondents’ resources of urban air pollution were also investigated in this study. As shown in Figure 2, most respondents reported that television or radio, internet sites and newspaper or magazine were the main sources for getting health-related knowledge about urban air pollution but less from doctor/physician or other health care professional.

## 4. Discussion

China has been going through a rapid development of urbanization and accelerated growth of its economy [30]. Many problems can be expected to occur at this stage, and urban air pollution is one of the most notable and challenging issues that must be faced [31,32]. As many major cities in China are undergoing an “urban reconstruction and modern industrialization” period, a substantial amount of infrastructure improvement has been undertaken. For instance, the fast-growing cities like Shanghai, Wuhan and Nanchang are racing to build subway systems that have attracted more migrant workers moving to these cities. Increased level of urban air pollution in these cities have placed the residents to more vulnerable adverse health condition: current researches have shown that many respiratory diseases werTABLEe due to air pollution, and long-term exposure to polluted air could damage people’s immune function [9,10,33,34,35].

This paper assessed the first steps in developing a model of the social dilemma of unsafe air quality in Chinese megacities and provided valuable insight to bridge the gap between scientific research and public health risk awareness of urban air pollution. Our study provides important theoretical references for air pollution risk management and decision-makers, as well as useful measures for risk prevention and reduction [36]. Understandings of the public perceptions of urban air pollution and related health risks and their influential factors are very important for the policymakers in their designing the relevant intervention programs [37]. On the other hand, it is critical for all individuals to rectify the misunderstanding of urban air pollution and related health risks, to enhance more comprehension and cooperation with environmental policy managements, and to lower the policy execution cost.

Our study showed that the public was generally satisfied with current air quality, however their anxious feelings about air pollution remained, especially among females and older adults. Zhang [31] reported that the females were particularly concerned about urban air pollution and related health risks, and ranked it in the first place. Our study also found that female expressed more negative and dissatisfied feelings when exposed to air pollutants. Kim et al. [32] showed that younger people had lower satisfaction levels with urban air quality; however we found that older respondents were less satisfied and more anxious. This disagreement could be due to the difference in general physical health condition between older adults and the younger. In addition, people who had a previous travel experience had lower satisfactions on local urban air quality because of their travel. In addition, public’s anxious feelings towards urban air pollution were also influenced by their health condition—healthier people were less anxious.

Previous studies showed that the public’s knowledge of air quality was associated with demographic characteristics [36,37,38]. Our study observed that educational level, income, health status and study sites were related to respondents’ understanding of air pollution related indicator (API). Those individuals with higher education, higher monthly income, and at good health status, had better knowledge about API. In addition, no differences of respondents’ general satisfaction and anxious feelings were found among the three study sites (Table 2, Table 3 and Table 5). However, the public’s knowledge of the API varies significantly among the three megacities (Table 4 and Table 5), which might be a reflection of variations in education and income level among these cities as shown in Table 1. This result is consistent with the findings reported in 1960s and some recent studies conducted in China [21,25,29].

Correlation analyses indicated weak positive correlations between respondents’ anxious feeling—general satisfaction, knowledge of API—general satisfaction and knowledge of API—anxious feeling. Hence, there is a need to establish the links between the urban air pollutant emissions and control, and public’s general feelings and attitudes toward the urban air quality and management actions through the investigation of public’s general satisfaction. It may veritably reflect and systematically understand the current situation of urban air quality management and its health related risk management from the angle of responders’ perception. This information could be very valuable for enhanced environmental management to local government. Since little was done in this study to explore the reasons why respondents’ general satisfaction, anxious feeling and knowledge of API toward current air quality attitudes correlated with each other, more in-depth investigation or open-ended questions survey to address this point needs to be designed and conducted in future.

There are many studies suggesting that the serious effects of poor air quality on the human respiratory system have become much more apparent [33,34,35]. Some studies [39,40] reveal that gender and individual’s health status significantly affect the public’s perceptions of air pollution and related health risks. It is widely believed that air pollution can cause or aggravate respiratory and lung diseases. Particularly, individuals who frequently suffer from asthma or respiratory diseases such as coughs are more sensitive to atmospheric pollutants, since these illnesses are triggered by air pollution. In this study, most megacity inhabitants believed that coughs/colds, eye problems and skin allergies were the three highest health risks due to urban air pollution, especially to the females and the older adults.

Current reports have seemed to be inconclusive regarding the relationship between the public risks perception and their income level. Some studies [41,42] showed that residents living in the well-developed regions were more prone to believe that poor air quality is responsible for their respiratory health, allergies and skin problems, while other studies [43,44] reported that those people with lower income were more likely to believe that there is no connection between poor air quality and health impact. Our study also showed that the public health concerns about air pollution were not affected by their income level. In addition, this study found that people with higher education level were more likely to believe the poor air quality may cause skin allergies, which is worthy of being properly addressed in the future.

Finally, when the responders were asked where they learned health-related knowledge about urban air pollution, most respondents reported television/radio, internet sites and newspapers/magazines to be the main sources. However, Bickerstaff et al.’s study [45] showed that very few people judged the quality through the air pollution related indicator released by the media. Even though people usually do not check the API specifically, reports and comments about air pollution problems through different media such as television, radio, internet sites, newspaper or magazine, etc., have increasingly impacted people’s awareness of urban air pollution and its adverse health impact. The diversified communication form, frequency of the reported information, the wording and the channel of promulgating such information can be the important factors affecting the public’s health-related knowledge, attitude and practice on urban air pollution.

Internet and new media like Weibo—the Chinese version of Twitter—and blogs greatly promote the flow and exchange for the information, and will amplify the effect of the environmental health-related risk (the so-called media amplifier effect) [46]. On the other hand, informal communication or conversation among the public is also an important way for people to apperceive outside health-related risks, which may function as an essential supplement to adjust people’s subjective feelings. Discussion and information exchange on air pollution between relatives, friends and colleagues could also play an influential role on people’s risk perception. This study shows that there are a quite a few respondents reporting that they obtained health-related knowledge about urban air pollution through their friends, family members, doctors, physicians or other health care professional. Findings from this study emphasizes the need for more frequent education campaigns of environmental health risks and protections in order to enhance the public’s self-protection consciousness as well as to monitor and improve air quality.

Despite its vigorous design, there are several limitations in this study. This survey was conducted at three megacities in China, and our inclusion criteria for research object is only for those who aged 18 years and above and living in the those megacities for at least one year, so the design of this study has limited participants from rural areas or temporary migrants. Thus, application of these findings to other areas and other population groups should be done with caution. Moreover, open-ended questions need to be designed to reveal new problems and deepen the understanding of the respondents’ attitudes towards current air quality in China’s megacities. For example, what kind of styles and education campaigns they want to enhance the public’s self-protection consciousness as well as to monitor and improve air quality. These are what need to be properly addressed in further research. In addition, social desirability of the participants may also have an effect on their answers since the results were self-reported from the interviewees.

## 5. Conclusions

This paper represents the first study addressing the public’s health risk awareness on urban air pollution among residents living in different megacities in China. The total sample size of 3868 provides powerful results for all cities. We found that: (1) female and older respondents had lower satisfaction and stronger feelings of anxiety about urban air quality; (2) people who had previous travel experience tended to report lower satisfaction with local urban air quality; (3) there were low positive correlations between respondents’ general satisfaction—anxious feelings, respondents’ general satisfaction—knowledge of API and knowledge of API—anxious feelings; (4) Most respondents believed that coughs/colds, eye problems and skin allergies were the three highest perceived health risks due to urban air pollution, especially females and older adults; (5) people with higher education levels were more likely to believe the poor air quality may cause skin allergies; (6) responders believed that television/radio, internet sites and newspaper/magazine are the main sources for the public to get health-related knowledge on urban air pollution.

Effective communication styles and public education are likely to increase the health risk awareness and understanding on urban air pollution for the individuals, families and community, and will encourage people to positively adapt preventive measures for the interest of safeguarding public health. There is also need for further research on the role of risk perception for behavior change through the interdisciplinary perspective to understand how public risk perceptions shape people’s behaviors.

## Figures and Tables

**Table 1:** Demographic information of the participants in three study sites (*n* = 3868).

Demographic Factors	Shanghai		Wuhan		Nanchang		Total	
	*n*	%	*n*	%	*n*	%	*n*	%
**Gender**								
Male	703	51.13	606	50.21	688	53.50	1997	51.63
Female	672	48.87	601	49.79	598	46.50	1871	48.37
**Age**								
<25	331	24.07	288	23.86	286	22.24	905	23.40
25–45	453	32.95	410	33.97	429	33.36	1292	33.40
45–65	412	29.96	362	29.99	385	29.94	1159	29.96
>65	179	13.02	147	12.18	186	14.46	512	13.24
Education level *								
≤Elementary School	93	6.76	139	11.52	138	10.73	370	9.56
Junior High–High School	720	52.37	628	52.03	659	51.24	2007	51.89
≥College	562	40.87	440	36.45	489	38.03	1491	38.55
Monthly income (RMB) *								
≤2999	266	19.35	291	24.11	308	23.95	865	22.36
3000–4999	578	42.04	522	43.25	552	42.92	1652	42.71
5000–7999	352	25.60	286	23.69	305	23.72	943	24.38
≥8000	179	13.02	108	8.95	121	9.41	408	10.55
Travel experience *								
Yes	1186	86.25	1008	83.51	975	83.83	3272	84.59
No	189	13.75	199	16.49	208	16.17	596	15.41
**Good health status**								
Yes	836	60.80	726	60.15	793	61.66	2355	60.88
No	539	39.20	481	39.85	493	38.34	1513	39.12
**Total**	1375	100	1207	100	1286	100	3868	100

* Chi-square significant at 5% level (for education level: χ^2^ = 21.471, *p* < 0.001; for monthly income: χ^2^ = 22.704, *p* = 0.010; for travel experience: χ^2^ = 7.602, *p* = 0.022).

**Table 2:** Respondents’ general satisfaction of current air quality.

Demographic Factors	Satisfactory		Unsatisfactory		χ^2^	*p*-Value
	*n*	%	*n*	%		
**Gender**					9.989	**0.002**
Male	928	46.47	1069	53.53		
Female	775	41.42	1096	58.58		
Age *					22.634	**<0.001**
<25	416	45.97	489	54.03		
25–45	580	44.89	712	55.11		
45–65	531	45.82	628	54.18		
>65	176	34.37	336	65.63		
**Education level**					0.013	0.993
≤Elementary School	162	43.78	208	56.22		
Junior High–High School	885	44.10	1122	55.90		
≥College	656	44.00	835	56.00		
**Monthly income (RMB)**					0.919	0.821
≤2999	389	44.97	476	55.03		
3000–4999	721	43.64	931	56.36		
5000–7999	408	43.27	535	56.73		
≥8000	185	45.34	223	54.66		
**Travel experience**					4.480	**0.034**
Yes	1417	43.31	1855	56.69		
No	286	47.99	310	52.01		
**Good health status**					0.761	0.383
Yes	1050	44.59	1305	55.41		
No	653	43.16	860	56.84		
**Anxious feelings**					142.55	**<0.001**
Yes	732	35.20	1348	64.80		
No	971	54.31	817	45.69		
**Knowledge of API**					31.15	**<0.001**
Yes	894	40.20	1330	59.80		
No	809	49.21	835	50.79		
**Study Sites**					0.464	0.793
Shanghai	602	43.78	773	56.22		
Wuhan	541	44.82	666	55.18		
Nanchang	560	44.55	726	56.45		
**Total**	1703	44.03	2165	55.97		

* Linear trend chi-square significant at 5% level (χ^2^ = 10.065, *p* = 0.002).

**Table 3:** Multivariable logistic regression analysis for the influencing factors among respondents’ satisfaction, anxious feelings and knowledge of API.

Variable(s)	Satisfaction (Unsatisfactory)		Anxious Feelings (Yes)		Knowledge of API (Yes)	
	OR	OR 95% CI	OR	OR 95% CI	OR	OR 95% CI
**Gender** (Male = control group)						
Female	1.26 **	1.07–1.38	1.51 *	1.22–2.01	1.02	0.89–3.31
**Age** (<25 = control group)						
25–45	1.30 *	1.15–1.48	1.33	0.93–1.97	2.06	0.83–2.38
45–65	1.28 *	1.23–2.06	1.48 **	1.12–3.25	1.56	0.77–3.26
>65	1.59 **	1.66–3.08	2.09 **	2.18–4.22	0.97	0.33–5.07
**Education level** (≤Elementary School = control group)						
Junior High–High School	1.12	0.79–2.13	1.07	0.88–2.30	1.31 **	3.22–8.91
≥College	1.29	0.97–3.07	0.99	0.71–4.06	2.89 **	5.81–13.07
**Monthly income (RMB)** (≤2999 = control group)						
3000–4999	1.29	0.90–1.67	1.21	0.64–1.77	1.17 *	1.05–4.32
5000–7999	1.58	0.78–1.36	1.79	0.19–3.02	2.51 **	3.57–7.27
≥8000	2.02	0.98–3.31	1.35	0.38–1.21	3.06 **	2.26–9.07
**Travel experience** (Yes = control group)						
No	0.78 *	0.35–0.71	1.07	0.17–1.09	1.21	0.21–1.13
**Good health status** (Yes = control group)						
No	1.46	0.61–1.27	2.11 **	3.01–5.06	1.37 *	1.05–4.11
**Study Sites** (Shanghai = control group)						
Wuhan	1.03	0.79–3.01	0.92	0.71–2.01	0.81 *	0.07–0.16
Nanchang	1.11	0.84–2.97	0.87	0.77–1.79	0.76 *	0.10–0.09

* *p*-value < 0.05, ** *p*-value < 0.01.

**Table 4:** Respondents’ feelings of anxiety when exposed to contaminated air.

Demographic Factors	Yes		No		χ^2^	*p*-Value
	*n*	%	*n*	%		
**Gender**					5.739	**0.017**
Male	886	44.37	1111	55.63		
Female	902	48.21	969	51.79		
Age *					14.734	**0.002**
<25	407	44.97	498	55.03		
25–45	581	44.97	711	55.03		
45–65	523	45.13	636	54.87		
>65	277	54.10	235	45.90		
**Education level**					0.436	0.804
≤Elementary School	177	47.84	193	52.16		
Junior High–High School	923	45.99	1084	54.01		
≥College	688	46.14	803	53.86		
**Monthly income (RMB)**					1.161	0.762
≤2999	401	46.36	464	53.64		
3000–4999	761	46.07	891	53.93		
5000–7999	428	45.39	515	54.61		
≥8000	198	48.53	210	51.47		
**Travel experience**					0.879	0.348
Yes	1502	45.90	1770	54.10		
No	286	47.99	310	52.01		
**Good health status**					61.438	**<0.001**
Yes	970	41.19	1385	58.81		
No	818	54.06	695	45.94		
**Knowledge of API**					13.372	**<0.001**
Yes	816	49.21	828	50.79		
No	972	40.20	1252	59.80		
**Study sites**						
Shanghai	626	45.53	749	54.47	1.581	0.454
Wuhan	576	47.72	631	52.28		
Nanchang	586	45.57	700	54.43		
**Total**	1788	46.23	2080	53.77		

* Linear trend chi-square significant at 5% level (χ^2^ = 6.844, *p* = 0.009).

**Table 5:** Respondents’ knowledge of air pollution related indicator (API).

Demographic Factors	Yes		No		χ^2^	*p*-Value
	*n*	%	*n*	%		
**Gender**					0.443	0.506
Male	859	43.01	1138	56.99		
Female	785	41.96	1086	58.04		
**Age**					0.259	0.967
<25	389	42.98	516	57.02		
25–45	550	42.57	742	57.43		
45–65	492	42.45	667	57.55		
>65	213	41.60	299	58.40		
Education level *					10.174	**0.006**
≤Elementary School	133	35.95	237	64.04		
Junior High–High School	842	41.95	1165	58.05		
≥College	669	44.87	822	55.13		
Monthly income (RMB) *					60.627	**<0.001**
≤2999	305	35.26	560	64.74		
3000–4999	662	40.07	990	59.93		
5000–7999	452	47.93	491	52.07		
≥8000	225	55.15	183	44.85		
**Travel experience**					1.439	0.230
Yes	1404	42.91	1868	57.09		
No	240	40.27	356	59.73		
**Good Health Status**					6.060	**0.014**
Yes	964	40.93	1391	59.07		
No	680	44.94	833	55.06		
**Study Sites**					8.610	**0.013**
Shanghai	625	45.45	750	54.55		
Wuhan	481	39.85	726	60.15		
Nanchang	538	41.84	748	58.16		
**Total**	1644	42.50	2224	57.50		

* Linear trend chi-square significant at 5% level (for education level: χ^2^ = 9.443, *p* = 0.002; for monthly income: χ^2^ = 59.593, *p* < 0.001).

**Table 6:** Correlation coefficients (ϕ) between respondents’ general satisfaction, anxious feeling and knowledge of API toward current air quality.

Variables	General Satisfaction	Anxious Feeling	Knowledge of API
General satisfaction	1	--	--
Anxious feeling	0.192 **	1	--
Knowledge of API	0.091 **	0.059 **	1

** *p* < 0.01.

**Table 7:** Respondents’ beliefs about the three highest health risks due to urban air pollution.

Demographic Factors	Cough/Cold				Eye Problem				Skin Allergies			
	*n*	%	χ^2^	*p*-Value	*n*	%	χ^2^	*p*-Value	*n*	%	χ^2^	*p*-Value
**Gender**			5.368	**0.021**			38.963	**<0.001**			8.672	**0.003**
Male	1341	67.15			1164	58.29			929	46.52		
Female	1321	70.60			1272	67.98			959	51.26		
**Age**			49.627	**<0.001**			8.728	**0.033**			0.404	0.940
<25	554	61.22			534	59.01			447	49.39		
25–45	891	68.96			823	63.70			635	49.15		
45–65	813	70.15			742	64.02			559	48.23		
>65	404	78.91	44.377 *	**<0.001**	337	65.82	6.915 *	**0.009**	247	48.24	0.344 *	0.558
**Education level**			2.160	0.340			0.914	0.633			101.871	**<0.001**
≤Elementary School	243	65.68			228	61.62			134	36.22		
Junior High–High School	1395	69.51			1256	62.58			879	43.80		
≥College	1024	68.68	0.275 *	0.601	952	63.85	0.906 *	0.341	875	58.69	97.863 *	**<0.001**
**Monthly income (RMB)**			0.771	0.856			1.180	0.758			1.305	0.728
≤2999	597	69.02			553	63.93			433	50.06		
3000–4999	1137	68.83			1041	63.01			795	48.12		
5000–7999	641	67.97			594	62.99			455	48.25		
≥8000	287	70.34	0.011 *	0.917	248	60.78	0.898 *	0.343	205	50.25	0.024 *	0.878
**Good health status**			6.461	**0.011**			1.888	**0.016**			2.198	0.138
Yes	1585	67.30			1463	62.12			1127	47.86		
No	1077	71.18			973	64.31			761	50.29		
**Study Sites**			1.298	0.523			1.527	0.466			8.685	**0.013**
Shanghai	962	69.96			882	64.15			715	52.00		
Wuhan	823	68.19			746	61.81			568	47.06		
Nanchang	877	68.20			808	62.83			605	47.05		
**Total**	2662	68.82			2436	62.98			1888	48.81		

* Those values represent linear trend chi-square.

## References

[1] Hajime A. (2003). Global air quality and pollution. Science.

[2] Mage D., Ozolins G., Peterson P., Webster A., Orthofer R., Vandeweerd V. (1996). Urban air pollution in megacities of the world. Atmos. Environ..

[3] Chan C.K., Yao X. (2008). Air pollution in mega cities in China. Atmos. Environ..

[4] Helfand W.H., Lazarus J., Theerman P. (2001). Donora, Pennsylvania: An environmental disaster of the 20th century. Am. J. Public Health.

[5] Davis D.L., Bell M.L., Tony F. (2002). A look back at the London smog of 1952 and the half century since. Environ. Health Perspect..

[6] World Health Organization (WHO) Ambient (Outdoor) Air Quality and Health. http://www.who.int/mediacentre/factsheets/fs313/en/.

[7] World Health Organization (WHO) (2006). Air Quality Guidelines for Particulate Matter, Ozone, Nitrogen Dioxide and Sulfur Dioxide.

[8] Samet J.M., Dominici F., Curriero F.C., Coursac I., Zeger S.L. (2001). Fine particulate air pollution and mortality in 20 U.S. cities, 1987–1994. N. Engl. J. Med..

[9] Pei D. (2011). Study on the association between ambient air pollution and daily cardiovascular and respiratory mortality in an urban district of Beijing. Int. J. Environ. Res. Public Health.

[10] Künzli N., Kaiser R., Medina S., Studnicka M., Chanel O., Filliger P., Herry M., Horak F., Puybonnieux-Texier V., Quénel P. (2000). Public-health impact of outdoor and traffic-related air pollution: A European assessment. Lancet.

[11] Arku R.E., Vallarino J., Dionisio K.L., Willis R., Choi H., Wilson J.G., Hempgill C., Agyei-Mensah S., Spengler J.D., Ezzati M. (2008). Characterizing air pollution in two low-income neighborhoods in Accra, Ghana. Sci. Total Environ..

[12] Baumbach G. (2012). Air Quality Control: Formation and Sources, Dispersion, Characteristics and Impact of Air Pollutants—Measuring Methods, Techniques for Reduction of Emissions and Regulations for Air Quality Control.

[13] Walters R. (2014). Air pollution and invisible violence. Invisible Crimes and Social Harms.

[14] Gurjar B.R., Jain A., Sharma A., Agarwal A., Gupta P., Nagpure A.S., Lelieve J. (2010). Human health risks in megacities due to air pollution. Atmos. Environ..

[15] National Bureau of Statistic of the People’s Republic of China China National Statistics Yearbook. http://www.stats.gov.cn/tjsj/ndsj/.

[16] Ministry of Environmental Protection of the People’s Republic of China China Environmental Bulletin in 2014. http://jcs.mep.gov.cn/hjzl/zkgb/?COLLCC=214580716&.

[17] Chen Z., Wang J.N., Ma G.X., Zhang Y.S. (2013). China tackles the health effects of air pollution. Lancet.

[18] Huang J., Zhang W., Zhu X., Gilliam F.S., Chen H., Lu X., Mo J. (2015). Urbanization in China changes the composition and main sources of wet inorganic nitrogen deposition. Environ. Sci. Pollut. Res..

[19] Xia T.Y., Wang J.Y., Song K., Da L.J. (2014). Variations in air quality during rapid urbanization in Shanghai, China. Landsc. Ecol. Eng..

[20] Bickerstaff K., Walker G. (2003). The place(s) of matter: Matter out of place—Public understandings of air pollution. Prog. Hum. Geogr..

[21] Elliott S.J., Cole D.C., Krueger P., Voorberg N., Wakefield S. (1999). The power of perception: Health risk attributed to air pollution in an urban industrial neighbourhood. Risk Anal..

[22] Chinese Law & Government (2009). 4th Press Conference on the Fourteenth Stage of Atmospheric Pollution Control Work in Beijing Municipality. Chin. Law Gov..

[23] Egondi T., Kyobutungi C., Ng N., Muindi K., Oti S., van de Vijver S., Ettarh R., Rocklöv J. (2013). Community Perceptions of Air Pollution and Related Health Risks in Nairobi Slums. Int. J. Environ. Res. Public Health.

[24] Vangeli P.A., Koutsidou A. (2014). Public perception for monitoring and management of environmental risk: The case of the tires’ fire in Drama region, Greece. J. Risk Res..

[25] Wang R., Yang Y., Chen R., Kan H., Wu J., Wang K., Maddock J.E., Lu Y. (2015). Knowledge, Attitudes, and Practices (KAP) of the Relationship between Air Pollution and Children’s Respiratory Health in Shanghai, China. Int. J. Environ. Res. Public Health.

[26] Wakefield S.E., Elliott S.J., Eyles J.D., Cole D.C. (2006). Taking environmental action: The role of local composition, context, and collective. Environ. Manag..

[27] Peptenatu D., Pintilii R.D., Draghici C. (2011). Environmental risk management of urban growth poles regarding national importance. Int. J. Environ. Sci. Technol..

[28] Muindi K., Egondi T., Kimani-Murage E., Rocklov J., Ng N. (2014). “We are used to this”: A qualitative assessment of the perceptions of and attitudes towards air pollution amongst slum residents in Nairobi. BMC Public Health.

[29] Zhang L., Yuan Z., Maddock J.E., Zhang P., Jiang Z., Lee T., Zou J., Lu Y. (2014). Air quality and environmental protection concerns among residents in Nanchang, China. Air Qual. Atmos. Health.

[30] Zhou Y., Ma L.J.C. (2015). China’s urbanization levels: Reconstructing a baseline from the fifth population census. China Q..

[31] Zhang J. (1994). Environmental hazards in the Chinese public’s eyes. Risk Anal..

[32] Kim M.H., Yi O.H., Kim H. (2012). The role of differences in individual and community attributes in perceived air quality. Sci. Total Environ..

[33] Fossati S., Metruccio F., Urso P., Ruggeri L., Ciammella M., Colombo S., Cerchiello M., Mauro A., Tibiletti M., de Vito G. (2006). Effects of short-term exposure to urban particulate matter on cardiovascular and respiratory systems—The PM-CARE study. Epidemiology.

[34] Zhang H., Ye Y., Diggle P., Shi J. (2008). Joint modeling of time series measures and recurrent events and analysis of the effects of air quality on respiratory symptoms. J. Am. Stat. Assoc..

[35] Gül H., Gaga E.O., Döğeroğlu T., Özden Ö., Ayvaz Ö., Özel S., Güngör G. (2011). Respiratory Health Symptoms among Students Exposed to Different Levels of Air Pollution in a Turkish City. Int. J. Environ. Res. Public Health.

[36] Howel D., Moffatt S., Prince H., Bush J., Dunn C.E. (2002). Urban air quality in north-east England: Exploring the influences on local views and perceptions. Risk Anal..

[37] Omanga E., Ulmer L., Berhane Z., Gatari M. (2014). Industrial air pollution in rural Kenya: Community awareness, risk perception and associations between risk variables. BMC Public Health.

[38] Liao X., Tu H., Maddock J.E., Fan S., Lan G., Wu Y., Lu Y. (2015). Residents’ perception of air quality, pollution sources, and air pollution control in Nanchang, China. Atmos. Pollut. Res..

[39] Badland H.M., Duncan M.J. (2009). Perceptions of air pollution during the work-related commute by adults in Queensland, Australia. Atmos. Environ..

[40] Shi X. (2015). Factors influencing the environmental satisfaction of local residents in the coal mining area, China. Soc. Indic. Res..

[41] Brody S.D., Peck B.M., Highfield W.E. (2004). Examining localized patterns of air quality perception in Texas: A spatial and statistical analysis. Risk Anal..

[42] Ferreira S., Akay A., Brereton F., Cuñado J., Martinsson P., Moro M., Ningal T.F. (2012). Life satisfaction and air quality in Europe. Ecol. Econ..

[43] Onkal-Engin G., Demir I., Hiz H. (2004). Assessment of urban air quality in Istanbul using fuzzy synthetic evaluation. Atmos. Environ..

[44] Fang M., Yao C.X. (2009). Managing air quality in a rapidly developing nation: China. Atmos. Environ..

[45] Bickerstaff K., Walker G. (2001). Public understandings of air pollution: The “localization” of environmental risk. Glob. Environ. Chang..

[46] Vijaykumar S., Jin Y., Nowak G. (2015). Social media and the virality of risk: The risk amplification through media spread (RAMS) model. J. Homel. Secur. Emerg. Manag..

